# The public health CARES initiative: a framework for public health education in high schools using the SMAART model

**DOI:** 10.3389/fpubh.2025.1563971

**Published:** 2025-07-10

**Authors:** Ashish Joshi, Niharika Jha, Michelle Taylor, Nichole Saulsberry-Scarboro, Courtney Orians, Sally Parish, Kristle Hodges Johnson, Lori Ward, Coree Entwistle, Satish Kedia, David Russomanno

**Affiliations:** ^1^School of Public Health, University of Memphis, Memphis, TN, United States; ^2^Shelby County Health Department, Memphis, TN, United States; ^3^Admissions Dual Enrollment and Special Enrollment Programs, University of Memphis, Memphis, TN, United States; ^4^Vice Provost and Director of Schools, University of Memphis, Memphis, TN, United States; ^5^University High School, University of Memphis, Memphis, TN, United States; ^6^Office of the Provost and Academic Affairs, The University of Memphis, Memphis, TN, United States

**Keywords:** public health education, secondary education, experiential learning, SMAART model, youth empowerment, social determinants of health (SDOH), health equity

## Abstract

Using the SMAART model (Sustainable, Multisector, Accessible, Affordable, Reimbursable, and Tailored), the Public Health CARES [Coping, Adaptability, Resilience, Empathy, and Success, (or PH-CARES)] initiative aims to address health inequities among youth to promote health education in schools and communities. PH-CARES integrates public health curriculum into high schools to enhance students’ knowledge and skills, fostering their adaptability, resilience, empathy, and success. The PH-CARES initiative includes the establishment of a public health dual-enrollment program, Public Health Clubs, and the Public Health Hackathon, all designed to provide hands-on learning experiences. Our preliminary assessments from these initiatives reveal that they bridged didactic learning to real-world applications and facilitate students’ engagement with public health campaigns, community projects, and case studies, while providing the opportunity to earn college credits through a dual-enrollment program. Students participating in these initiatives gained a deeper understanding of public health issues, population health, social determinants, and data analytics, and were sensitized to local health issues preparing them for future public health careers. The initiative’s holistic approach demonstrates the potential for scalable public health education programs that empower students and promote long-term community health and well-being, establishing a model that can be implemented globally.

## 1 Introduction

### 1.1 Public health interventions for youth

Youth across the globe are experiencing many pressing public health issues, including mental health disorders, obesity, vaping, the opioid crisis, cyberbullying, institutionalized racism, social isolation, and climate change, to name a few (1–3). Adolescents and young adults are prone to risk-taking behavior, such as substance use, unprotected sex, and reckless driving, which may result in higher morbidity and mortality. Similarly, long-term effects of unhealthy diet, lack of physical activity, and excessive use of the internet and social media could be consequential in terms of poor physical and mental health (4). Some sections of the population are particularly vulnerable to environmental toxins and adverse social conditions, including racism, violence, and poor living and working situations (5, 6). Many of the factors that contribute to morbidity and mortality among adolescents are preventable (2, 7). Policymakers and practitioners have long recognized that adolescence is a critical phase in which to enhance understanding of good health, safety, and general well-being (2, 8). Interventions aimed at educating, influencing, and incentivizing youth to adopt protective health behaviors are crucial.

It is not easy to bring about sustained and effective improvements in behavioral and community health (9, 10). Public health professionals are tasked with both educating vulnerable populations and promoting environmental modifications to reduce exposure to risks while facilitating healthy behavior and public safety (9). Some interventions may be implemented as stand-alone programs, but many others need to be delivered in tandem to have synergistic effects on the overall well-being of adolescents. Previous research has found that health outcomes vary considerably at the individual level; therefore, interventions focused on behavioral change at the individual level have limited impact (9, 11). Public health campaigns often emphasize behavioral change at the community level by enforcing positive health messages to promote overall health improvement and maintenance. However, when considering interventions for the adolescents, the temporality of the campaign becomes critical, as improving health behaviors earlier in life may lead to long-term positive health outcomes (2, 10, 12).

### 1.2 Social determinants of health

Social determinants of health (SDOH) are influences stemming from outside the clinical realm that profoundly affect individuals’ health in the short- and long-term (13). Social determinants are related to the environments where people spend their time, including home, neighborhood, and workplace, and cultural and societal forces that influence the conditions of daily life (14). The literature has consistently shown that SDOH has a powerful influence on health outcomes, outweighing lifestyle, healthcare access, and genetic factors. For example, in one study, patients with lupus indicated that SDOH were responsible for 30–55% of their health outcomes (15). Social determinants also affect sleep duration and quality, education quality, diet behavior, hygiene, stress, and anger, all of which contribute to individuals’ health and well-being, especially among young people (5, 16). There is a growing recognition that health behaviors are influenced by system-level factors as well as day-to-day choices people make. SDOH are also closely linked to health disparities and inequities, and there is an increasing consensus in the public health community that addressing SDOH is critical to attaining greater health equity (14).

The Healthy People 2030 initiative emphasizes addressing SDOH as one of its five overarching goals to improve “upstream” factors related to the economic, social, and physical environments in which people are immersed on a daily basis (14). Traditionally, public health work has focused on collaborating with multisectoral partners such as education, transportation, and housing, for instance, to address disparities and improve human health (17). Therefore, community engagement is a critical component of effective public health practice, especially to address social determinants of health (18).

### 1.3 Need for public health workforce

In 1920, CE Winslow defined public health as “the science and art of preventing disease, prolonging life, and promoting health through the organized efforts and informed choices of society, organizations, public and private communities, and individuals” (19). As such, the work of public health is aimed at establishing and maintaining systems to prevent and mitigate the effects of infectious and chronic disease and improve the health of all people (19). Public health is a multidisciplinary practice, requiring expertise from a variety of STEM fields, including epidemiologists, biostatisticians, informaticians, microbiologists, and data scientists, as well as social and behavioral scientists (20). Moreover, critical skills for effective public health work include problem-solving, critical thinking, communication, innovation, and collaboration. Public health inherently requires an interdisciplinary, team-based approach (21). The current public health workforce is fragmented and lacks necessary skills; thus, collaboration and recruitment from a variety of medical and non-medical fields are increasingly vital. Addressing the health, social, and environmental threats of our times demands innovative, comprehensive, and coordinated efforts (22).

In the United States (US), the Public Health Workforce Interests and Needs Survey (PH-WINS) found that nearly half (46%) of state and local public health employees left their current positions during the five-year study period (23, 24). Resignation rates were particularly high among younger public health staff, with those under the age of 35 being much more likely to leave their positions (24). Between 2010 and 2013, the public health workforce experienced a significant loss to the tune of approximately 40,000 jobs; the workforce capacity has not increased since that time, even during the COVID-19 pandemic (24). Findings from the PH-WINS survey, released in March 2022, found that the governmental public health workforce had high levels of reported burnout, which contributed to an intent to leave, especially during and following the pandemic (24). Additionally, the survey identified the need for greater policy engagement and additional training on justice, equity, diversity, and inclusion as being critical to maintaining the public health workforce (23). The WHO’s Action Plan for Enhancing Public Health Services and Capacities includes strengthening the public health workforce as a key element of future success for societal wellbeing (25).

Recent analysis of public health job postings indicates a high demand for basic workplace skills such as cross-sectoral communication and leadership, as well as project development, implementation, and management skills (26, 27). Among those already in leadership or management positions, analysis found that financial skills were identified as the most common training need (23). Multiple quantitative analyses of the PH-WINS data and other studies have agreed that certain skill sets are critical for success in the public health field, including persuasive communication skills, information analytics, problem-solving, systems thinking, and cultural sensitivity (28, 29). Higher education is rapidly adjusting its approach to meet the demands of employers who expect their employees to take on greater responsibility and have a greater breadth of skills than previously anticipated (30, 31). As such, leadership and management, financial competencies, and informatics are usually prioritized in current public health curricula (23, 27). This rapidly evolving landscape indicates a need to frequently assess and adapt public health curriculum to ensure that higher education equips students with skills relevant to the contemporary workforce and current and emerging public health challenges (31). One of the most important qualities that employers look for in an employee is their ability to apply theoretical knowledge in a real-world setting (32); thus, the trend toward experiential learning in higher education is an important shift to increase the employability of public health students (33).

There is a need for strong and flexible leaders who are trained in developing cross-sector partnerships to initiate effective interventions that address SDOH across the lifespan (25). To progress toward better outcomes and health equity, communities need timely access to comprehensive and actionable health data. It is equally important for public health projects to have clear metrics to document and assess the impact of their efforts in order to develop evidence-based interventions for the future, including those addressing SDOH and unique needs of adolescents (8, 14). It is imperative to train and develop public health leaders for tomorrow; for this reason, enhancing public health literacy and integrating a public health curriculum into high schools is an important step in the right direction (34).

This paper aims to describe an innovative and multi-faceted “Public Health CARES (PH-CARES)” framework for public health education in high school settings using the SMAART model. This framework advances health equity and sustains positive health and well-being among adolescents. The PH-CARES framework incorporates five key pillars: Coping, Adaptability, Resilience, Empathy, and Success, to inform, educate, intervene, evaluate, communicate, and disseminate public health information among high school students. PH-CARES is implemented through a wide range of interrelated activities, including academics, research, entrepreneurship, and storytelling, to train PH-CARES youth ambassadors who can facilitate health equity and enhance community health and well-being across local and global (i.e., glocal) settings.

## 2 Pedagogical framework: the SMAART model and the Public Health in Action (PHIA) curriculum

### 2.1 PH-CARES framework using the SMAART model

**Figure 1:**
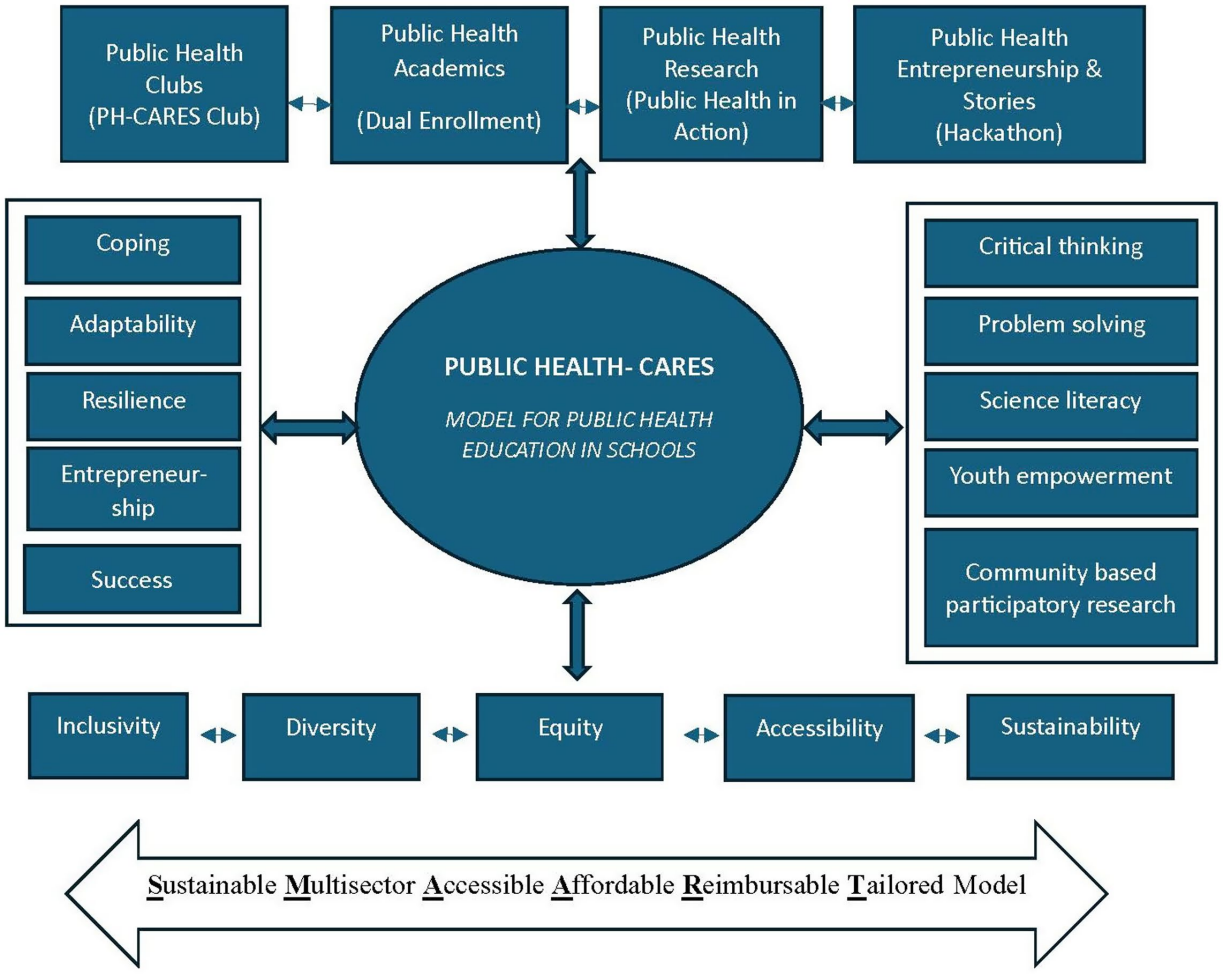
Adapting the SMAART model to implement the PH-CARES initiative toward establishing public health education in high schools.

The leadership at the University of Memphis School of Public Health has designed and launched a new initiative, “PH-CARES,” (Figure 1) an innovative model for public health education in high schools (35). The initiative is built around the previously utilized SMAART model (Sustainable, Multisectoral, Accessible, Affordable, Reimbursable, and Tailored) to design, develop, and implement human-centered solutions toward enhancing well-being and promoting improved health outcomes for individuals, families, and communities (36).

### 2.2 Perspective on the SMAART model

The SMAART model is a Population Health Informatics framework utilizing several information and learning theories that can be applied toward the development of an intelligent, applicable, and evidence-based model for presenting a public health curriculum in schools (36). The Data, Information, and Knowledge (DIK) pathway is applied to help organize the pertinent information in a meaningful way (37–39). The model seeks to develop person-centered interfaces that focus on task requirements and functionality through iterations of design solutions while promoting multidisciplinary teamwork (38–40). Graphic displays are integrated into the program following Cognitive Fit Theory (CFT) to engage both auditory and visual learning processes (41, 42), while Information Processing Theory guides the organization of information into meaningful units (43, 44). Ensuring that the information presented matches the task and the comprehension of the learner will further create a satisfying educational experience and outcomes. Following behavioral and humanistic theories of learning, the SMAART model aspires to develop a curriculum that is interconnected, relevant, nimble, adaptable to multiple formats, and responsive to feedback. This initiative aims to sensitize youth about the field of public health education, provides a window into public health career opportunities, and broadens students’ understanding of health and well-being at the individual, community, and global levels.

### 2.3 Public health education through Public Health in Action (PHIA)

Public Health in Action (PHIA) is an experiential learning approach designed to guide students through a unique pathway in the field of public health. PHIA provides rigorous preparation for advanced public health education for students planning to enter multidisciplinary public health programs and other health disciplines, such as health informatics and health policy. Through PHIA, high schoolers can develop skills that will help them effectively interpret public health information and data and accurately apply this knowledge to address societal problems in a real-world context. The experiential PHIA format also cultivates a sense of social responsibility and empowerment to engage with and address the health needs of their communities. Examples and experiences from students’ daily lives are incorporated to advance their understanding of interconnectivity among public health issues, i.e., the relationship between social factors, personal behavior, and disease transmission. PHIA also introduces students to essential skills that are in high demand in the public health workforce and provides opportunities to practice effective communication, collaboration, and critical thinking in an experiential learning environment. Giving students a chance to experience the spark of ingenuity that comes from collaborative problem solving may inspire them to further explore and evolve their understanding of the relevance of public health in their own lives and in society at large. The modules covered under the PHIA curriculum focus on competencies, skills, and knowledge frequently desired by employers for entry-level public health jobs (27).

Using the WHO Framework of Global Competency and Outcomes, we categorized public health functions and skills into 6 domains in the PHIA Experiential Learning curriculum (Table 1) (45). This curriculum emphasizes collaboration between the high schools and local community organizations. The goal is to build a bridge between theory and practical applications so that students develop a more complete understanding and familiarity with public health issues. The PHIA curriculum increases students’ understanding of public health and its challenges and assists them in acquiring skills and knowledge to design, develop, and implement innovative solutions to address public health issues using a human-centered design approach.

**Table 1:** Structure of the PHIA curriculum modules and matching the current public health jobs with this course’s contents.

PHIA modules	Descriptions	WHO-based global PH competencies	Public health skills
Public health 101	Introduces key concepts, principles, and practices related to public health.	Community centeredness Decision-making Collaboration Personal conduct	Public health communication and campaigns—outreach/intervention Public health education and promotion Infectious and chronic disease prevention and control Environmental—industrial hygiene and occupational safety Public health leadership
Importance of research in public health	Students explore the role of research in public health practice, its ethical considerations, and various research methodologies used in the field.	Evidence-informed practice Decision-making	Public health education and promotion Technical writing, grant writing, reports, and manuscripts Data collection and analysis Documentation and reporting of project activities Problem solving and critical thinking Literature review and reporting
Data gathering techniques in public health	Introduces various methods and tools used for collecting, analyzing, and interpreting data in public health research and practice.	Evidence-informed practice Communication	Data collection and analysis Software competency—MS Office and data analysis software Develop and conduct surveys Qualitative and quantitative research methods and analysis
Story telling with public health data	Students learn how to transform complex data into accessible and impactful stories that engage diverse audiences, raise awareness, and drive action towards improving population health.	Communication Collaboration	Public health communication and campaigns—outreach/intervention Public health education and promotion Technical writing, grant writing, reports, and manuscripts Multi-agency/cross-sectoral communication Quantitative research methods and statistical analysis Communication—using media for health promotion
Role of data, information and knowledge in public health	Explore the interplay between data, information, and knowledge in shaping public health policies, programs, and interventions.	Evidence-informed practice Decision-making Communication	Public health communication and campaigns—outreach/intervention Public health education and promotion Technical writing, grant writing, reports, and manuscripts Health Program evaluation and improvement Problem solving and critical thinking
Human centered design approaches in public health	Students acquire skills to design, develop and implement user centric public health interventions to promote health equity.	Community-centeredness Collaboration	Conduct user needs assessment and stakeholder engagement Develop and implement behavior change intervention plan Evidence-based intervention and best practices
Evaluation of public health intervention	Provide students with an understanding of evaluation methods and techniques used to assess the effectiveness, efficiency, and impact of public health programs and interventions.	Community-centeredness Decision-making	Administrative and organizational skills Documentation of project activities Health Program evaluation and improvement Environmental—health planning, investigation, and assessment Public health leadership Evidence-based intervention and best practices Needs assessment
Technological innovations in public health	Explain role of emerging technologies in transforming public health practice, research, and policy.	Collaboration Decision-making	Health technology interventions and innovations Health informatics—data management and visualization Experience with electronic health records
Public health entrepreneurship	Focuses on development, implementation, and sustainability of innovative solutions to address public health issues.	Collaboration Decision-making	Project development, implementation, management, and compliance Administrative and organizational skills Collaboration and partnerships Public health leadership Financial management, budget, grant management
Public health is “Global”	Teaches students about the interconnectedness of global and local factors in shaping public health outcomes and interventions.	Collaboration Personal conduct	Cultural competency—sensitivity to population-specific issues Collaboration and partnerships Environmental—industrial hygiene and occupational safety Food and water safety concerns
Case-studies	Provide students with opportunities to analyze real-world public health issues, interventions, and policies through case study analysis.	Personal conduct Decision-making	Cultural competency—sensitivity to population-specific issues Project development, implementation, management, and compliance Case management skills—referrals and assessments

Two unique components of this curriculum that enhance students’ abilities to apply public health concepts using a collaborative approach are: (1) students engage in a team-based collaborative learning activity in the classroom or online, which enables them to develop interpersonal communication and negotiation skills. To complete the tasks provided, they work together, explore areas of interest, accommodate ideas, and navigate personal differences; (2) students participate in experiential learning activities in a real-life public health service environment or research setting in which they work closely with practitioners or researchers to address a public health issue. PHIA curriculum has four broad goals: (a) to broaden students’ exposure to public health, (b) to facilitate critical thinking and develop analytical, communication, and teamwork skills essential for addressing public health challenges, (c) to synthesize public health skills such as learning how to gather public health data, present and visualize information in a way that is meaningful and insightful, and aid in evidence-informed decision-making, and (d) to experience first-hand the public health practice and/or research. Tables 1, 2 outline the various modules, their brief descriptions, associated WHO-based global public health competencies, and public health skills that students acquire in this curriculum.

**Table 2:** PHIA experiential learning assessments.

*Components of experiential learning*
Classroom-based case studies *Integration in curriculum*: Incorporate at least 4 case studies into the PHIA curriculum, reflecting current public health issues worldwide. *Interactive learning*: Use interactive methods like group discussions, role-playing, and problem-solving activities. *Assessment*: Evaluate students’ understanding and analytical skills through exams, written reports, and presentations. Community-based projects *Project identification*: Collaborate with local health organizations to identify 3 community-based public health projects. *Student involvement*: Engage students in these projects through roles such as data collection, community outreach, and health education. *Mentorship*: Assign public health professionals as mentors to guide students throughout the projects. Field visits and observations *Site visits*: Organize visits to local health departments, hospitals, and community health centers. *Observation*: Allow students to observe public health professionals at work and understand various public health functions and operations. *Reflection*: Require students to submit reflection journals detailing their observations and learning experiences. Workshops and seminars *Expert sessions*: Host workshops and seminars led by public health experts on topics such as epidemiology, project implementation, and evaluation. *Hands-on activities*: Include activities such as study/project design, literature review and analysis and implementation plan. *Interactive Q&A*: Facilitate Q&A sessions to allow students to engage directly with experts and clarify their doubts. Public health practice *Community engagement*: Focus on engaging different community groups, including schools, local businesses, and residential areas. *Impact assessment*: Measure the impact of these activities through surveys and community feedback.
*Outcomes and benefits*
*Enhanced learning*: Students gain practical skills and a deeper understanding of public health issues. *Community impact*: Local communities benefit from health campaigns and projects conducted by students. *Professional development*: Students develop essential skills such as critical thinking, teamwork, and problem-solving. *Career awareness*: Increased awareness and interest in public health careers among students.

In addition, the PHIA curriculum is designed to introduce students to the skills necessary to succeed in today’s public health workforce (26, 27). Key areas of proficiency include public health communication, which requires competence with basic computer programs, data analysis tools, documentation, and reporting skills, along with public health knowledge. Cultural competency, multi-agency communication capacity, project management, and program evaluation skills are also in high demand. Some public health positions demand case management, data collection, health informatics, technical writing, policy development, and leadership skills as well. Specialized areas of public health practice and research demand specific skill sets, which are also encouraged in the curriculum, such as financial management, problem-solving, survey development, quantitative and qualitative research, and disaster preparedness.

## 3 Learning environment: components of the PH-CARES initiative

### 3.1 Public health academics through dual enrollment

A dual enrollment program has been designed and implemented to provide 11th and 12th graders with an opportunity to experience a higher level of public health curriculum while earning college credits toward a bachelor’s degree in public health (BSPH) at the University of Memphis. Participation in public health courses also exposes high school students to a wide range of career opportunities in the public health field and shows them that they have the potential to offer effective solutions to today’s health issues by pursuing a career in public health. Students who choose to attend the University of Memphis and declare a major in Public Health have the option to transfer dual enrollment credits toward their bachelor’s degree. The 18-credit curriculum emphasizes both practical and theoretical skills and can set students well along the path to a BSPH. Classes in the dual enrollment program include:

- *Population health and society*: Introduces students to the field of population health and examines the role of major social, economic, behavioral, and environmental factors in communities and populations while applying evidence-based knowledge to improve population health outcomes among socially disadvantaged groups.
- *Environmental and climate health*: Introduces the framework, methodologies, and applications of environmental health and public health impacts and adaptations to climate change while equipping students with approaches to assess and control environmental risks and climate threats.
- *Social determinants and health disparities*: Introduces students to the social and economic conditions that affect individual and population health, as well as the role of public health practitioners in improving health outcomes through interventions and policy change.
- *Health data analytics and informatics*: Provides an overview of data sources, flow, management, and analysis of health data, focusing on describing, presenting, and interpreting empirical evidence in public health.
- *Global health crises and milestones*: Introduces students to significant milestones in the field of public health, taking a global perspective when analyzing and exploring major public health events that have affected health and well-being worldwide.

The Public Health CARES dual enrollment initiative was launched in 2023 and taught by faculty members from the University of Memphis School of Public Health, assisted by graduate students who help facilitate discussions and activities. The program is currently implemented in nine (*n* = 9) high schools, reaching an average of approximately 51 students per semester (Fall 2023 = 71, Spring 2024 = 34, Fall 2024 = 25, Spring 2025 = 76). The participating schools include a mix of urban and rural institutions in the mid-South, with the city of Memphis as the central hub. Student demographics reflect the diverse socioeconomic and ethnic backgrounds of the area. The program follows a semester-based schedule, aligning with the academic calendar of participating high schools.

### 3.2 PH-CARES club

The PH-CARES clubs provide opportunities for students to actively contribute to the betterment of their own well-being and participate in enhancing the health of their communities. Club meetings are facilitated by public health specialists who guide students to imagine and enact creative solutions to common public health issues at both local and global levels. The clubs implement a wide range of activities, including organizing interactive public health workshops for peers and the larger community, celebrating public health days, taking part in community-based participatory research, and meaningful interpretation of the health data they collect. These activities challenge students to develop analytical skills, brainstorm ideas, and implement public health campaigns to inform their communities about public health issues. The public health clubs utilize an experiential learning approach through the Public Health in Action (PHIA) curriculum. The curriculum is designed by combining the basics of public health concepts and theories with health research, data, design thinking, and the application of public health interventions, innovations, and entrepreneurship to solve real-world public health challenges. Students in the public health club also have the opportunity to participate in the Hackathon.

### 3.3 Public health entrepreneurship and storytelling through hackathons

The goal of this initiative is to facilitate a gathering of young people who are eager to explore the pressing public health issues in their surroundings and present them with an opportunity to brainstorm and collaborate to develop solutions for these challenges both locally and globally. This innovative initiative aims to inspire Leadership and Educational Advancement among youth to solve Public Health (LEAP) problems. The experiential learning component fosters a deeper comprehension and mastery of public health constructs and skills, as well as provides an opportunity to work with peers to find creative answers for real-world public health problems (34–36, 38, 39). The hackathon engages youth and young adults to collaborate, brainstorm, and work on practical solutions for critical community health issues. This initiative allows participants to practice actuating their ideas, achieve their objectives, and contribute innovative and feasible solutions to public health problems, empowering students to become real public health heroes.

## 4 Implementation and scalability plans

The implementation plan for PH-CARES follows a structured, four-phase approach to enhance public health education and community well-being:

1. *Phase 1: landscape analysis*: The project begins with a detailed landscape analysis to identify public health needs and skills gaps in the local and regional community, which guides the subsequent curriculum development.
2. *Phase 2: curriculum pilot*: The curriculum, which integrates theoretical knowledge with practical applications, is piloted in designated high schools. This phase includes hands-on experiential learning activities such as community-based projects, field visits, and expert-led workshops.
3. *Phase 3: well-being model development*: A well-being model is designed, piloted, and refined based on gathered health data. This model focuses on interventions in mental health, nutrition, physical activity, and preventive care.
4. *Phase 4: scaling and sustaining interventions*: The final phase involves scaling and sustaining interventions across more schools and community centers, ensuring long-term impact and continuous improvement in program engagement and public health outcomes.

This ambitious 24-month implementation plan emphasizes collaboration, continuous feedback, and iterative refinement to achieve its goals effectively.

PH-CARES is intended as a scalable model. However, effective replication necessitates careful adaptations to local contexts. Following the implementation plan above, educational leaders must consider essential factors such as the geographic location of institutions, the trained educators, institutional preparedness, and synchronization with local academic schedules. To date, elements of the PH-CARES framework are being successfully developed and implemented in India, Saudi Arabia, Taiwan, Turkey, and Portugal, as well as in the United States (46). These initiatives have met with success by aligning educational modules with local and national public health priorities and linguistic requirements. The efficacy of the Public Health Hackathons further substantiates the generalizability of this program, as three annual hackathons have already been conducted, with the last one in 2025 attracting 89 students from 16 interdisciplinary teams. These activities exemplify robust youth interaction in varied contexts and provide a reproducible model for participatory learning. The PH-CARES framework, by maintaining adaptability to local context while upholding fundamental learning objectives, has potential for wider application in various global settings.

Overall, the implementation of these PH-CARES initiatives addresses a variety of public health issues in high schools and the broader community. By identifying key areas where students and community members face health challenges, the PHIA approach facilitates the planning and implementation of strategies that are creative, practical, and evidence-based to promote good health and well-being. This multifaceted approach to high school-level public health education can lead to a scalable and sustainable well-being model that can be implemented locally and globally, fostering healthier and more resilient communities.

## 5 Evaluation plan

Evaluations of the PH-CARES initiative involve a mixed-methods approach, combining pre- and post-program surveys to assess changes in student knowledge, attitudes, and interest in public health careers, along with qualitative methods such as open-ended feedback to capture student experiences and insights. Planned outcome measures include (1) improvement in student knowledge and understanding of public health concepts through pre- and post-program surveys, (2) student engagement and satisfaction with learning experiences, and (3) follow-up tracking of students’ interest in public health careers and academic pathways. These metrics will help determine the program’s short-term educational impact and long-term potential in shaping future public health leaders. Specific assessment strategies for each component are as follows.

### 5.1 Dual enrollment assessment

We utilized a blend of informal and formative evaluation methodologies to evaluate the efficacy of the PHIA curriculum across many areas of student learning and engagement. Classroom assessments encompassed observable measures of active participation in discussions, as well as the completion and quality of assignments. We also received feedback on students’ perceptions of instructional quality, content delivery, and instructor responsiveness. Initial findings indicated robust student involvement and favorable feedback about collaborative and creative problem-solving activities. We are assessing what adjustments should be made to the course syllabi to best adapt to the dual enrollment setting and how to support instructors and high school staff to ensure the success of the program. Future assessments will be guided by the qualitative feedback collected in these initial courses and will include a formal evaluation using quantitative surveys to measure learning outcomes and expectations of the students and teachers. Outcome measures for the dual enrollment program will include assessments of students’ academic performance as well as student feedback about their experience in the classroom. Aside from the traditional grading rubric, future assessments will gauge public health knowledge acquisition, critical thinking, teamwork competencies, and engagement, including course completion and attendance rates. Additional measures to be collected in quantitative surveys include feedback on course content, instructional/pedagogical style, instructor’s motivation, inclusivity, and feedback quality.

### 5.2 Public health clubs assessment

Public Health Club leaders have collected informal student feedback, reflective journals, and field notes that recorded club dynamics and tracked club activities. Club leaders are also part of the liaison with community partners who host the club for hands-on outreach activities and have collected informal feedback from the process of establishing those relationships. Preliminary data indicates that students involved in the club express heightened awareness of public health issues, critical thinking, and a greater interest in public health careers. The informal nature of club involvement provides club leaders with a special insight into the students’ motivations and engagement styles, which will be instructive in tailoring the club activities going forward. Qualitative input received from students and club leaders thus far will be used to shape future club activities and outreach efforts to align with both student interest and community needs. Ultimately, the intent of the club is to provide students real-world public health experiences and inspire them to pursue greater understanding and involvement in public health issues. We also plan to develop a brief quantitative survey that students can complete at the end of each semester to supplement information collected by club leaders.

### 5.3 Public health hackathon assessment

Instructors who oversaw the activities of the Public Health Hackathon teams provided qualitative feedback on observable measures of student engagement with the four stages of the hackathon: problem identification, idea generation, project design, and project pitching. Judges’ scoring templates and notes have also been collected for the evaluation purposes. The scoring template considers how well hackathon projects demonstrated theoretical quality, applicable design, capacity to meet a demonstrable public health need, clarity of presentation, and inspiration. Generally, students engaged in the hackathon showed strong abilities to apply theoretical knowledge to practical issues and demonstrated that they perceived the significance of public health in relation to both their personal lives and professional goals. Some of the most valuable components emerging from the hackathon thus far are the opportunities for intensive teamwork and the immediate and thorough feedback from established public health professionals. The informal data gathered from instructors’ and judges’ records will be used to create a quantitative assessment tool. In the future, participating students will be invited, though not required, to provide qualitative feedback about their experience in the Hackathon using this tool.

## 6 Discussion

As a model of public health education in high schools, PH-CARES has significant implications for public health education, community well-being, and long-term health outcomes. First, integrating public health curriculum into high school settings prepares students with critical knowledge and skills essential for addressing contemporary health challenges (34). This early exposure to public health concepts not only enhances students’ understanding of health issues but also inspires potential career interests in the health sector, contributing to a more informed and health-conscious future workforce (2, 47). Additionally, high schoolers’ engagement at the community level fosters stronger collaboration between educational institutions and other important community organizations, such as health departments (35). This synergy enhances the effectiveness of public health interventions and ensures they are tailored to the community’s specific needs (47). By providing practical, hands-on experiential learning, the PHIA curriculum also empowers students to become active participants in promoting and sustaining public health within their communities.

Providing public health education opportunities in high school familiarizes students with relevant health information, expands opportunities to learn and practice organizational, leadership, and problem-solving skills, and normalizes positive health behaviors that could impact personal and community health outcomes for years to come (34, 35). Practical-level health education has the potential to yield exponential benefits, as research has demonstrated a strong association between academic outcomes and health behaviors (48, 49). On the other hand, the risky health behaviors among adolescents, such as poor diet and physical activity and the use of tobacco, alcohol, and other substances, are often associated with poor academic outcomes (48, 49). Given the benefits of integrating public health into high school curricula, we surmise that high schoolers should have exposure to public health information, which will empower them with a foundational understanding of population health and community-based participatory research (34, 49).

Provision of an effective public health curriculum will also fortify the role of high schools in the community, as it will demonstrate how schools can participate in reducing adolescent health risks, connect students to essential public health information and resources, engage parents and other community stakeholders, and foster positive relationships between high schoolers and the adults in their lives (34, 35). A well-designed and implemented school-based public health program can help adolescents make sense of their world as they approach adulthood and utilize these skills to thrive during their newfound independence. It is also a crucial step toward raising awareness of and appreciation for various public health issues and crises we collectively face, with the hope that new generations can engage in devising solutions to build a healthier world for all.

While the PH-CARES model demonstrates promise for broader dissemination, its successful implementation in diverse global contexts requires careful consideration of feasibility. Potential challenges may include differences in educational systems, limited access to trained instructors, varying levels of public health infrastructure and internet capability, and the need for culturally and contextually relevant curriculum adaptation. Furthermore, achieving educator buy-in and building strong institutional partnerships will be critical for effective delivery. To maintain program fidelity and ensure quality implementation, the PH-CARES model and its associated materials should not be replicated without formal collaboration and authorization from the program developers. Use of these materials requires prior written permission and formal collaboration with the program’s developers.

## 7 Conclusion

PH-CARES is a dynamic and interactive approach for integrating public health education into schools and represents a transformative initiative for public health education and community well-being. By incorporating an active and experiential public health curriculum, developing hands-on experiential learning opportunities, and implementing targeted human-centered community health and well-being initiatives, PH-CARES addresses both the educational and practical needs of the community while cultivating tomorrow’s public health workforce. The implementation of the PH-CARES initiative as a well-being model in schools and community centers has the potential to address a wide range of health issues, from mental health to chronic disease prevention, leading to improved quality of life and long-term health for students and residents. This holistic approach benefits individual participants and builds a more resilient and health-literate community, setting a precedent for expanding these initiatives in other regions.

These initiatives empower students with essential public health skills, foster a deeper understanding of health issues, and inspire future careers in public health, addressing gaps in the existing public health workforce. This multifaceted approach, involving collaboration between schools, health departments, and community organizations across local and global settings, ensures that interventions are tailored and effective, resulting in measurable improvements in health outcomes. By actively exposing students to public health information and related careers at a younger age, this approach encourages the next generation to contribute meaningfully to their communities’ health and well-being. Ultimately, PH-CARES creates an accessible pathway for a healthier, more informed, and engaged community, demonstrating the critical role of education and collaboration in advancing public health.

## Data Availability

The original contributions presented in the study are included in the article/supplementary material, further inquiries can be directed to the corresponding author.
